# Community Development between *Porphyromonas gingivalis* and *Candida albicans* Mediated by InlJ and Als3

**DOI:** 10.1128/mBio.00202-18

**Published:** 2018-04-24

**Authors:** Maryta N. Sztukowska, Lindsay C. Dutton, Christopher Delaney, Mark Ramsdale, Gordon Ramage, Howard F. Jenkinson, Angela H. Nobbs, Richard J. Lamont

**Affiliations:** a Department of Oral Immunology and Infectious Diseases, University of Louisville School of Dentistry, Louisville, Kentucky, USA; b Bristol Dental School, University of Bristol, Bristol, United Kingdom; c School of Medicine, Nursing and Dentistry, University of Glasgow, Glasgow, United Kingdom; d Biosciences, College of Life and Environmental Sciences, University of Exeter, Exeter, United Kingdom; KUMC

**Keywords:** *Candida albicans*, *Porphyromonas gingivalis*, RNA-Seq, microbial communities, polymicrobial synergy

## Abstract

The pleiomorphic yeast Candida albicans is a significant pathogen in immunocompromised individuals. In the oral cavity, C. albicans is an inhabitant of polymicrobial communities, and interspecies interactions promote hyphal formation and biofilm formation. C. albicans colonizes the subgingival area, and the frequency of colonization increases in periodontal disease. In this study, we investigated the interactions between C. albicans and the periodontal pathogen Porphyromonas gingivalis. C. albicans and P. gingivalis were found to coadhere in both the planktonic and sessile phases. Loss of the internalin-family protein InlJ abrogated adhesion of P. gingivalis to C. albicans, and recombinant InlJ protein competitively inhibited interspecies binding. A mutant of C. albicans deficient in expression of major hyphal protein Als3 showed diminished binding to P. gingivalis, and InlJ interacted with Als3 heterologously expressed in Saccharomyces cerevisiae. Transcriptional profiling by RNA sequencing (RNA-Seq) established that 57 genes were uniquely upregulated in an InlJ-dependent manner in P. gingivalis-C. albicans communities, with overrepresentation of those corresponding to 31 gene ontology terms, including those associated with growth and division. Of potential relevance to the disease process, C. albicans induced upregulation of components of the type IX secretion apparatus. Collectively, these findings indicate that InlJ-Als3-dependent binding facilitates interdomain community development between C. albicans and P. gingivalis and that P. gingivalis has the potential for increased virulence within such communities.

## IMPORTANCE

Many diseases involve the concerted actions of microorganisms assembled in polymicrobial communities. Inflammatory periodontal diseases are among the most common infections of humans and result in destruction of gum tissue and, ultimately, in loss of teeth. In periodontal disease, pathogenic communities can include the fungus Candida albicans; however, the contribution of C. albicans to the synergistic virulence of the community is poorly understood. Here we characterize the interactions between C. albicans and the keystone bacterial pathogen Porphyromonas gingivalis and show that coadhesion mediated by specific proteins results in major changes in gene expression by P. gingivalis, which could serve to increase pathogenic potential. The work provides significant insights into interdomain interactions that can enhance our understanding of diseases involving a multiplicity of microbial pathogens.

## INTRODUCTION

Periodontitis is a common inflammatory disease which affects the integrity of the tissues that surround and support the teeth. Around half the adult population in the United States experiences some form of the disease, and periodontitis is the sixth most common infection worldwide (1, 2). Additionally, periodontitis and periodontal pathogens are associated with serious systemic conditions such as rheumatoid arthritis, atherosclerosis, and some forms of cancer (3–5). Periodontitis ensues from the action of complex heterogeneous microbial communities that inhabit the subgingival compartment (6). Within those communities, organisms can collectively regulate physiological activities, and microbial constituents have developed functional specialization (7–9). Keystone pathogens, such as Porphyromonas gingivalis, can raise community pathogenic potential (or nososymbiocity) (10, 11). Accessory pathogens such as Streptococcus gordonii which are considered commensal alone can increase the pathogenicity of P. gingivalis (11). Indeed, P. gingivalis and S. gordonii interact through physical attachment and chemical-mediated communication (12), and dual-species communities are more pathogenic in animal models of periodontal diseases than either organism is alone (13).

In addition to bacteria, fungi represent a significant component of the oral microbiome (14). *Candida* species such as C. albicans are common inhabitants of the oral cavity and colonize polymicrobial biofilm communities (15). Specific interactions have been identified between C. albicans and a range of bacteria, e.g., *Pseudomonas*, *Staphylococcus*, and *Streptococcus* (16–23). There is evidence to suggest that these interactions may modulate the clinical course of infection and have an impact on treatment regimens (19, 24–29). Furthermore, interspecies interactions are considered important in development of denture stomatitis (30) and, potentially, also periodontal disease (31, 32). In subjects with chronic periodontitis, the rate of C. albicans carriage can increase, together with higher isolation frequencies of periodontal bacterial pathogens such as P. gingivalis (32). P. gingivalis can increase hyphal formation by C. albicans (33), and the organisms can coinhabit polymicrobial biofilms *in vitro* (34, 35). In addition, C. albicans can enhance invasion of gingival epithelial cells by P. gingivalis (36). However, the nature of the interaction between P. gingivalis and C. albicans has yet to be investigated in molecular detail. In this study, we examined the bacterial and fungal adhesins that mediate coadhesion and the influence of interspecies binding on the transcriptome of P. gingivalis.

## RESULTS

### P. gingivalis and C. albicans interactions.

**FIG 1:**
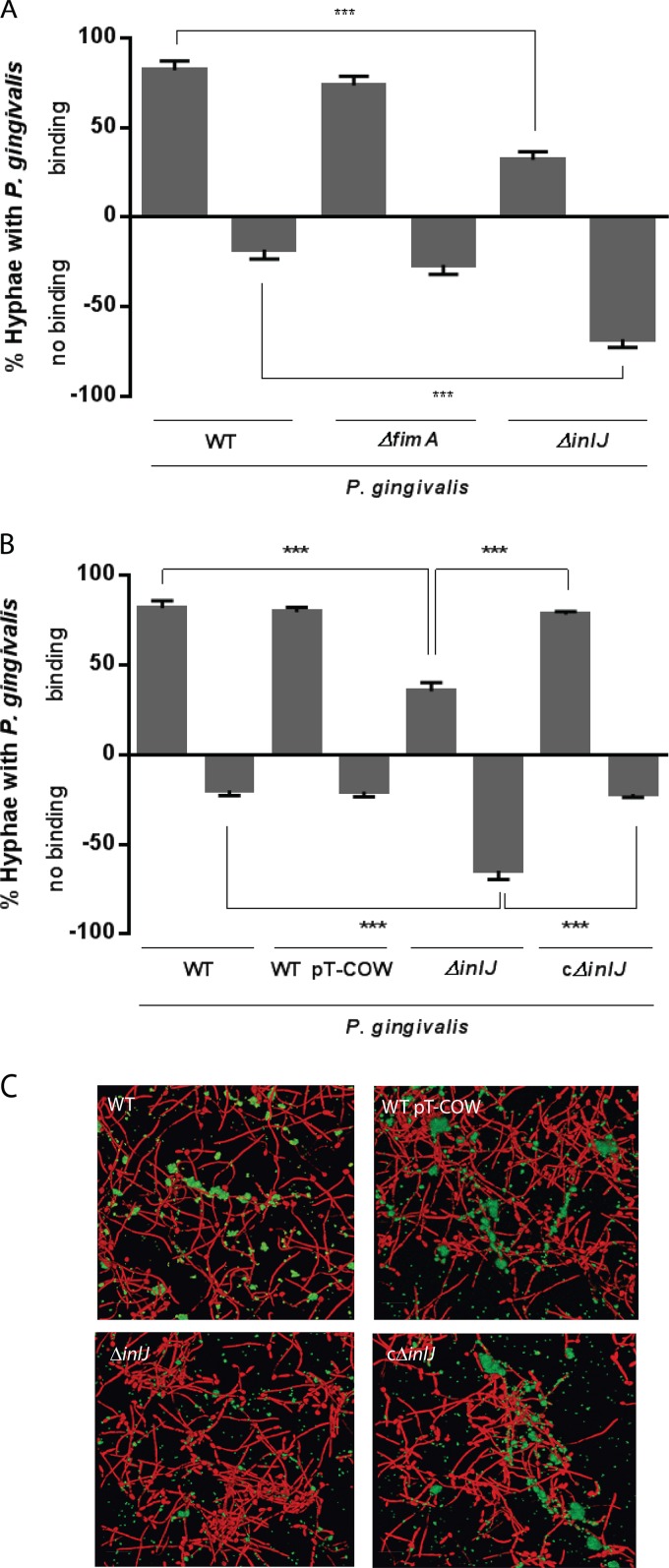
P. gingivalis interacts with C. albicans in an InlJ-dependent manner. (A) Percentages of total C. albicans SC5314 hyphae with attached cells of P. gingivalis 33277 (WT), Δ*fimA*, or Δ*inlJ* strains were determined on the basis of the following binding results: binding, including extensive attachment of bacteria to hyphae with bacteria clumping and bacterial cells aligned along hyphae in distinct patches, and no binding, including sparse or no interactions between bacteria and hyphae. One hundred hyphae were counted for each pairing. Results are representative of 4 independent experiments and are expressed as means ± standard deviations of the means (SD); *n* = 4. ***, *P* < 0.001 (by analysis of variance [ANOVA] with Tukey *post hoc* test). (B) Interactions of C. albicans SC5314 with P. gingivalis 33277 (WT), 33277+pT-COW (WT pT-COW), the Δ*inlJ* mutant, or the *inlJ* mutant complemented with the *inlJ* gene in *trans* (cΔ*inlJ*). Percentages of hyphae with bacteria attached were calculated on the basis of bacterial binding level as described for panel A. Results are representative of 4 independent experiments and are expressed as means ± SD; *n* = 4. ***, *P* < 0.001 (by ANOVA with Tukey *post hoc* test). (C) Fluorescence confocal micrographs of C. albicans SC5314 biofilms (red, stained with hexidium iodide) formed on saliva-coated glass for 3 h with attached cells of P. gingivalis 33277 (WT), 33277+pT-COW (WT pT-COW), the Δ*inlJ* mutant, or the *inlJ* mutant complemented with the *inlJ* gene in *trans* (cΔ*inlJ*) (green, stained with FITC). Biofilms were imaged at ×63 magnification. Merged images present projections of z-stacks obtained with Volocity software and are representative of results of 3 independent experiments.

C. albicans is a persistent colonizer of the human oral cavity and a common constituent of subgingival biofilms (17, 37, 38). Therefore, we investigated the ability of P. gingivalis to adhere to C. albicans in suspension. As shown in Fig. 1A, P. gingivalis adheres to C. albicans hyphae, and approximately 80% of hyphal filaments demonstrated a binding phenotype with P. gingivalis. In contrast, binding of P. gingivalis to yeast or pseudohyphal forms of C. albicans was rarely observed (see Fig. S1 in the supplemental material). Since the FimA component fimbriae of P. gingivalis are responsible for many adhesive properties of the organism (39, 40), we next examined the involvement of FimA in P. gingivalis interactions with candidal hyphae. A *fimA*-deficient mutant of P. gingivalis did not show a reduction in binding to C. albicans compared to the wild-type parental strain (Fig. 1A), indicating that other surface components of P. gingivalis mediate interspecies adherence. Previously, we had found that the InlJ internalin-family protein is required for optimal homotypic biofilm formation by P. gingivalis and is also involved in heterotypic biofilm control (41); thus, we tested the involvement of InlJ in P. gingivalis-C. albicans coadhesion. As shown in Fig. 1A, an isogenic *inlJ* mutant of P. gingivalis was significantly impaired in binding to C. albicans. To confirm the adhesion-mediating role of InlJ, a P. gingivalis strain was constructed in which the *inlJ* gene deletion was complemented with the wild-type *inlJ* allele expressed in *trans* from pT-COW (strain cΔ*inlJ*). Adherence of the cΔi*nlJ* mutant to candidal hyphae was restored to wild-type P. gingivalis levels (Fig. 1B), verifying the role of InlJ in mediating attachment of P. gingivalis to the hyphae of C. albicans. Screening mutants of P. gingivalis lacking minor fimbrial adhesin Mfa1 or hemagglutinin HagB found no effect on coadhesion with C. albicans (not shown), implicating InlJ as the predominant P. gingivalis adhesin in this interaction.

10.1128/mBio.00202-18.1 FIG S1  Examples showing minimal binding of P. gingivalis to yeast or pseudohhyphal forms of C. albicans. Fluorescently (FITC) labeled P. gingivalis cells were incubated with C. albicans cells for 1 h. Binding was visualized by phase-contrast microscopy (upper panels) and by fluorescence microscopy (lower panels). Download FIG S1, EPS file, 1.7 MB. Copyright © 2018 Sztukowska et al. 2018 Sztukowska et al. This content is distributed under the terms of the Creative Commons Attribution 4.0 International license.

### Role of InlJ in heterotypic biofilm formation.

As cells in biofilms can display phenotypes that are distinct from those seen with their free-living counterparts, we investigated early biofilm formation using C. albicans and P. gingivalis. C. albicans biofilm formation was initiated on saliva-coated glass coverslips, and, as shown in Fig. 1C, C. albicans cells attached to the surface and formed hyphal filaments to which P. gingivalis cells clearly bound. Adherence of P. gingivalis Δ*inlJ* to hyphal filaments was diminished, consistent with the planktonic condition. The complemented strain of P. gingivalis, the cΔ*inlJ* mutant, adhered to hyphal filaments under biofilm conditions at the same level as the wild type. These findings support the idea that InlJ is a mediator of P. gingivalis binding to C. albicans under both planktonic and sessile conditions.

### Inhibition of P. gingivalis interaction with C. albicans by InlJ.

**FIG 2:**
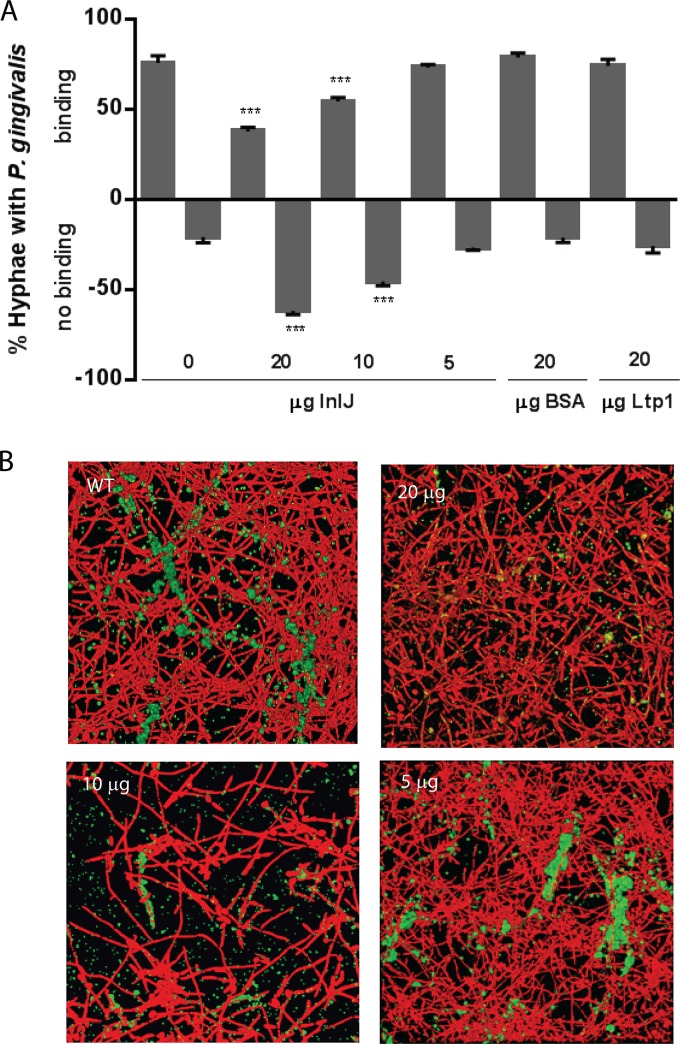
InlJ protein inhibits interaction of P. gingivalis with C. albicans. (A) Percentages of total C. albicans SC5314 hyphae with attached P. gingivalis 33277 in the presence of rInlJ protein at the concentrations indicated. Binding was calculated as described for Fig. 1. Recombinant P. gingivalis tyrosine phosphatase (Ltp1) and bovine serum albumin (BSA) (20 µg) were used as control proteins. Results are representative of 4 independent experiments and are expressed as means ± SD; *n* = 4. ***, *P* < 0.001 (compared to the control condition with no exogenous protein added by ANOVA with Dunnett’s correction). (B) Fluorescence confocal microscopy projections of C. albicans SC5314 biofilms (red, stained with hexidium iodide) formed on saliva-coated glass for 3 h with P. gingivalis 33277 (green, stained with FITC) in the presence of InlJ protein at the concentrations indicated. Biofilms were imaged at ×63 magnification. Merged images present projections of z-stacks obtained with Volocity software and are representative of results of 3 independent experiments.

To provide further insight into the role of InlJ in P. gingivalis-C. albicans coadhesion, recombinant protein was expressed as a His-tagged fusion and tested for inhibition of P. gingivalis binding to C. albicans. Figure 2A shows that recombinant InlJ (rInlJ) inhibited P. gingivalis binding in the suspension assay in a dose-dependent manner and that up to 65% inhibition was seen in the presence of 20 µg rInlJ. Control proteins bovine serum albumin (BSA) and rLtp1, an irrelevant phosphatase protein from P. gingivalis (42), did not display inhibitory activity. The same inhibitory effect was observed when dual-species biofilms were developed in the presence of rInlJ. P. gingivalis adherence to hyphae was reduced in a concentration-dependent manner in the presence of rInlJ protein (Fig. 2B). As relatively large amounts of soluble protein were required for inhibition, the presentation of InlJ on the bacterial surface may be necessary for the maintenance of optimal active structure. These findings support the model that InlJ mediates the attachment of P. gingivalis to C. albicans.

### Als3 is necessary for C. albicans interactions with P. gingivalis.

**FIG 3:**
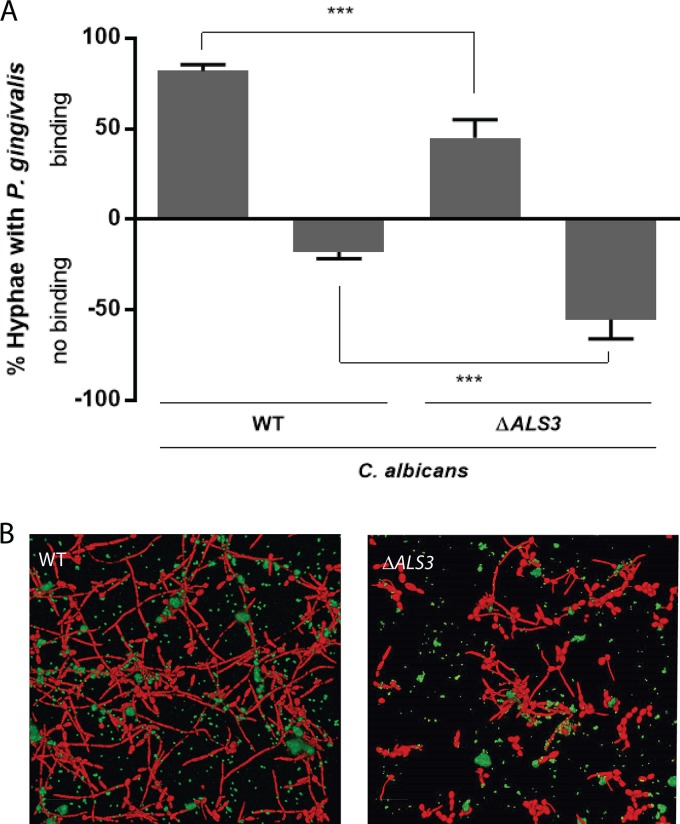
C. albicans Als3 is required for biofilm formation with P. gingivalis. (A) Percentages of total C. albicans UB1936 (WT) or mutant strain UB1930 (*als3*Δ) with attached P. gingivalis 33277 were calculated on the basis of bacterial binding levels as described for Fig. 1. Results are representative of 4 independent experiments and are expressed as means ± SD; *n* = 4. ***, *P* < 0.001 (by ANOVA with Tukey *post hoc* test). (B) Fluorescence confocal microscopy projections of biofilms of C. albicans UB1936 (WT) or mutant strain *als3Δ* (red, stained with hexidium iodide) formed on saliva-coated glass for 3 h with P. gingivalis 33277 (green, stained with FITC). Biofilms were imaged at ×63 magnification. Merged images present projections of z-stacks obtained with Volocity software and are representative of results of 3 independent experiments.

Previous studies demonstrated that the hypha-specific adhesin Als3 is important for C. albicans biofilm formation and for adhesion to host tissue and to the oral early plaque colonizer S. gordonii (43, 44). Further, Als3 is associated with hyphae (45), to which P. gingivalis preferentially binds. The potential involvement of Als3 in P. gingivalis binding was examined using an *als3*Δ *als3*Δ-*URA3* mutant (designated *als3*Δ). In the suspension assay, binding of C. albicans *als3*Δ to P. gingivalis decreased 58% (Fig. 3A). The *als3*Δ mutant formed sparse biofilms with few hyphae (Fig. 3B), consistent with the role of this protein in biofilm formation (46). Nonetheless, binding of P. gingivalis to the *als3*Δ mutant hyphae that were present was less than that seen with wild-type C. albicans. These results do not exclude the possible involvement of other Als proteins or, indeed, of other candidal adhesins, which may play a complementary or overlapping role in binding with Als3.

### InlJ interacts with Als3.

**FIG 4:**
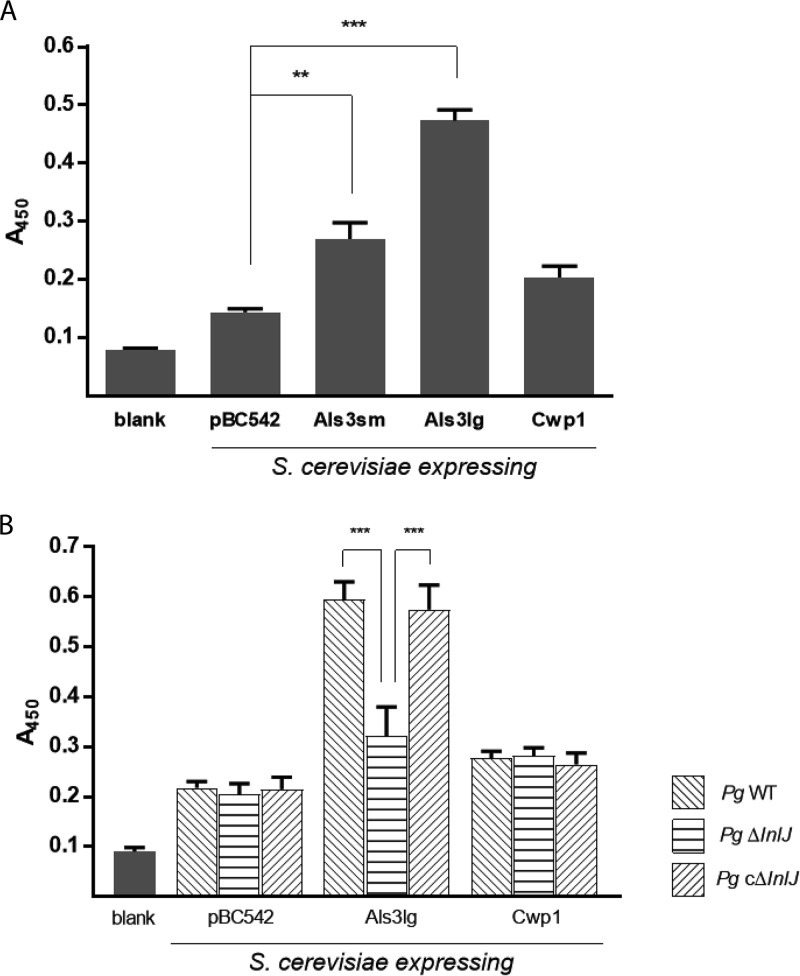
C. albicans Als3 interacts with InlJ of P. gingivalis. (A) Attachment of rInlJ protein to S. cerevisiae cells expressing candidal adhesins Als3sm, Als3lg, or Cwp1, or empty pBC542 vector was analyzed with an ELISA using His-tagged MAb (1:2,000). (B) Attachment of the P. gingivalis (*Pg*) WT strain, the Δ*inlJ* mutant, or the cΔ*inlJ* mutant to S. cerevisiae cells expressing candidal adhesins Als3lg or Cwp1, or empty pBC542 vector was analyzed with an ELISA using P. gingivalis antibodies (1:5,000). Results are representative of 3 independent experiments and are expressed as means ± SD; *n* = 3. **, *P* < 0.01; ***, *P* < 0.001 (by ANOVA with Tukey *post hoc* test).

Our findings suggested that C. albicans Als3 can act as a component of an adhesin-receptor system with P. gingivalis. To investigate whether Als3 interacts directly with InlJ, we utilized Saccharomyces cerevisiae cells expressing C. albicans adhesins. In an enzyme-linked immunosorbent assay (ELISA), rInlJ bound to S. cerevisiae cells expressing Als3 (derived from either the large or small *ALS3* allele) but not to control cells or cells expressing S. cerevisiae wall protein Cwp1 (Fig. 4A). Moreover, binding of P. gingivalis whole cells to S. cerevisiae expressing Als3 occurred only in the presence of InlJ (Fig. 4B). In control experiments, all strains of S. cerevisiae attached to the ELISA plates to the same degree (Fig. S2). These findings support a model whereby InlJ binds to hyphal Als3 to effectuate P. gingivalis-C. albicans association.

10.1128/mBio.00202-18.2 FIG S2  Control experiment showing equal levels of binding of engineered S. cerevisiae cells to ELISA plates. S. cerevisiae cells were deposited onto microtiter plates as described in Materials and Methods and detected using rabbit polyclonal Ab to S. cerevisiae (1: 10,000) followed by goat anti-rabbit HRP-conjugated IgG secondary antibodies (1:5,000). Results are representative of 3 independent experiments and are expressed as means ± SD; *n* = 3. Download FIG S2, EPS file, 1.2 MB. Copyright © 2018 Sztukowska et al. 2018 Sztukowska et al. This content is distributed under the terms of the Creative Commons Attribution 4.0 International license.

### Transcriptional profiling of InlJ-dependent P. gingivalis*-C.* albicans interactions.

**FIG 5:**
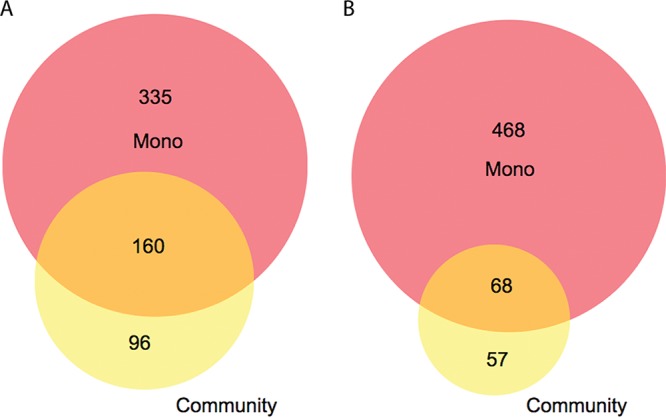
Venn diagram representing differentially expressed genes that were either (A) downregulated or (B) upregulated in the P. gingivalis 33277 WT strain compared to the Δ*inlJ* mutant, with and without coincubation with C. albicans. Yellow shading represents unique genes in coculture, red shading represents unique genes in monoculture (Mono), and orange shading represents genes that appeared under both conditions.

RNA sequencing (RNA-Seq) was utilized to examine the transcriptional responses of P. gingivalis in InlJ-dependent communities with C. albicans under planktonic coculture conditions. Comparing the P. gingivalis wild type to P. gingivalis Δ*inlJ* coincubated with or without *Candida*, 256 P. gingivalis genes in the parental strain were downregulated in the coincubation, with 96 of these being unique (i.e., not regulated without *Candida*) (Fig. 5A). Among those 96 genes, 58 had a Log2 fold change level of >1 (see Table S1 in the supplemental material). Overall, there were markedly fewer differences in expression under the coculture condition than under the monoculture condition, and there were over twice as many genes downregulated in the parental strain as in the InlJ-deficient cells (Table S1). Similarly, 125 genes were upregulated in the P. gingivalis wild type in the presence of C. albicans, with 57 of these being unique to the coincubation (Fig. 5B). Among those 57 unique genes, 21 had a Log2 fold change level of >1 (Table S1).

10.1128/mBio.00202-18.4 TABLE S1  Differentially regulated P. gingivalis gene analysis by RNA-Seq. Download TABLE S1, XLSX file, 0.3 MB. Copyright © 2018 Sztukowska et al. 2018 Sztukowska et al. This content is distributed under the terms of the Creative Commons Attribution 4.0 International license.

**FIG 6:**
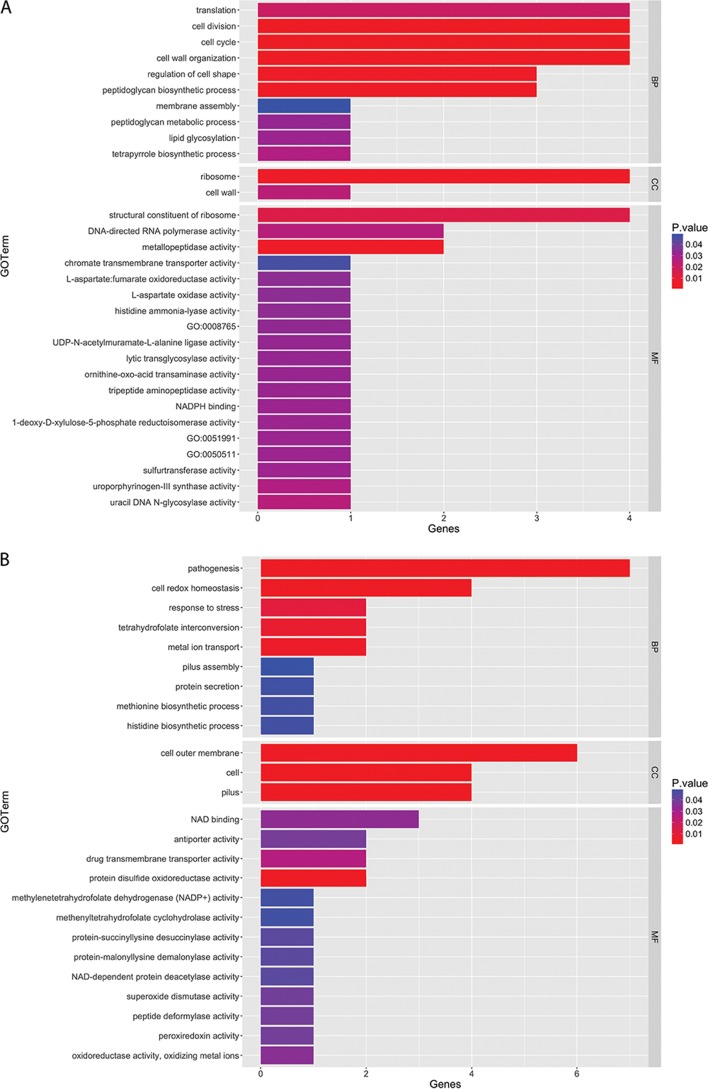
Distribution of genes that were significantly upregulated (A) and significantly downregulated (B) in the P. gingivalis 33277 (WT) strain relative to the Δ*inlJ* mutant in communities with C. albicans, grouped into the following Gene Ontology (GO) categories: biological process (BP), cellular component (CC), and metabolic function (MF). All terms have a *P* value of <0.05 based on results of the GOSeq hypergeometric distribution test.

**FIG 7:**
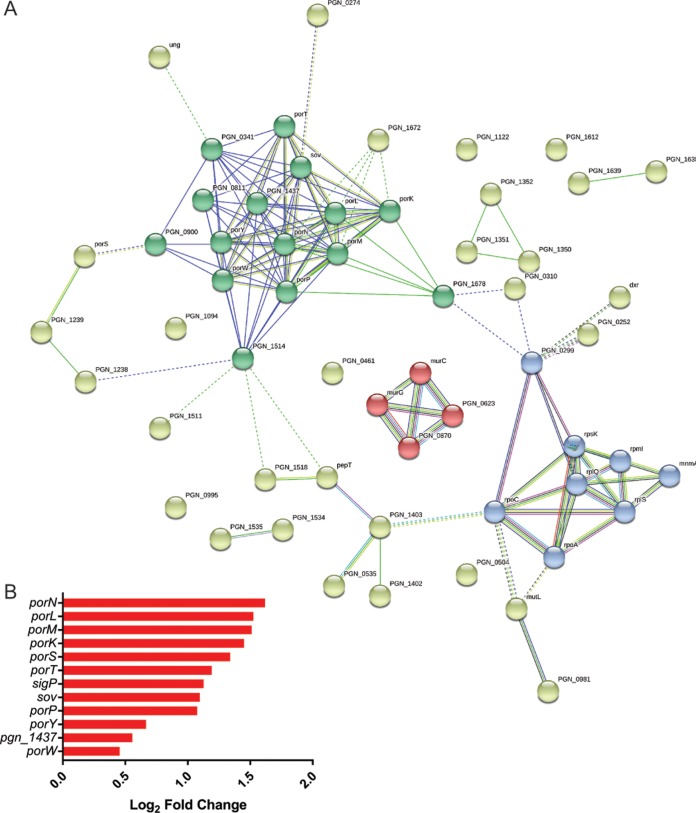
(A) STRING analysis of network of 3 groupings of related genes: "por" (genes corresponding to secretion systems), “rp” (genes corresponding to ribosomal proteins), and “mur” (corresponding to peptidoglycan biosynthesis). The genes indicated are those upregulated in P. gingivalis with InlJ in the context of a community shared with C. albicans. Edges between nodes represent protein interactions between nodes. The greater the number of edges, the larger the evidence base for identification of a functional link. These edges are drawn from curated databases (light blue), from experimental data (purple), and from predicted interactions (green, red, and blue). Other edges are also drawn and are from text mining, coexpression, and protein homology data. (B) Differential expression of T9SS genes in the P. gingivalis 33277 (WT) strain relative to the Δ*inlJ* mutant in response to C. albicans expressed as Log2 fold change. See Materials and Methods for statistical thresholds.

The 57 unique coculture InlJ-dependent upregulated genes were assigned to 31 Gene Ontology (GO) terms with an overenrichment *P* value of <0.05 (Fig. 6A). Among those 31 GO terms, biological process terms comprised 10, cellular component terms comprised 2, and molecular function terms comprised 19. Among the biological process terms, the most significantly overrepresented terms comprised cell wall organization terms, cell cycle terms, and cell division terms. The most significantly enriched cellular component terms were ribosome terms and cell wall terms, and terms corresponding to metallopeptidase activity, structural constituent of the ribosome, and uracil DNA N-glycosylase activity were those most significantly enriched among the molecular function terms. Collectively, these results suggest that InlJ-dependent association with C. albicans increases growth and division of P. gingivalis. Consistent with this, 3 genes involved in peptidoglycan biosynthesis, *murE*, *murC*, and *murG*, were upregulated with InlJ present, although only one of those genes, *murG*, had a Log2 fold change level of >1. An illustration depicting the results of a STRING network analysis (Fig. 7A) depicts genes corresponding to nodes, namely, ribosomal protein genes, peptidoglycan biosynthesis genes, and genes of the type IX secretion system (T9SS), according to k-means clustering, and also shows the potential for interactions among the products of these differentially regulated genes. There have been 18 components of the T9SS recognized in P. gingivalis to date, and the machinery is responsible for the translocation of over 30 proteins from the periplasm across the outer membrane (47–49). Targets of the T9SS include a number of virulence-associated proteins, including the gingipain proteases (47–49). Genes encoding 9 components of the T9SS machinery were upregulated in an InlJ-dependent manner; those genes included *porPKLMN*, representing an operon whose members are cotranscribed (Fig. 7B). The *porPKLMN* operon is controlled by the PorXY two-component system, which operates through S*igP*, an extracytoplasmic function (ECF) sigma factor. Genes encoding PorY and SigP were also upregulated by C. albicans. Although only two of the T9SS cargo proteins, PGN_1437 and the thiol protease PGN_0900, were upregulated (Table S1), C. albicans could potentially increase the pathogenicity of P. gingivalis indirectly through induction of type IX-dependent secretion of virulence factors, without affecting gene expression.

The 96 unique coculture InlJ-dependent downregulated genes were assigned to GO terms (Fig. 6B). Among those 96 genes, 25 showed overrepresentation (*P* value of <0.05). Of the 25 genes, 9 were part of the biological process GO category, with the term "pathogenesis" being the most highly represented followed by "cell redox homeostasis" and then "metal ion transport." Note that the data corresponding to the term "pathogenesis" were not P. gingivalis specific but were assembled from a variety of organisms. Overpopulation among the members of this category is derived from the genes for the fimbrial adhesins (*fimA* and *mfa1*) and the *hagA* gene encoding a hemagglutinin adhesin. Expression of these adhesion-associated genes may be upregulated in the mutant to compensate for the loss of InlJ. Data corresponding to the term "tetrahydrofolate interconversion" also showed significant regulation, and this pathway has been found to have a significant impact on the pathogenicity of P. gingivalis–S. gordonii dual-species communities (50). Within the cellular component category, 3 terms were significantly enriched from the upregulated genes, namely, the terms "cell outer membrane," "cell," and "pilus." The majority belonged to the molecular function category, with 13 terms being enriched. The term "protein disulfide oxidoreductase" was shown to be the most significantly overrepresented term, followed by "drug transmembrane transport activity."

## DISCUSSION

C. albicans is an opportunistic fungal pathogen which colonizes the gut, genital tract, and oral cavity of healthy individuals. Disruption of immune surveillance or broad-spectrum antibiotic therapy can allow overgrowth and realization of pathogenic potential by the organism. C. albicans can cause superficial infections, such as oropharyngeal candidiasis (thrush) and vulvovaginal candidiasis, and also life-threatening systemic infections (17, 51, 52). In addition, C. albicans biofilm infections are common on prosthetic devices such as urinary or intravascular catheters, artificial joints, and voice boxes (53). C. albicans is a pleiomorphic fungus and can transition among three distinct morphological states: yeast cells, pseudohyphae, and filamentous hyphae. Different morphological states are associated with colonization and growth, and the hyphal form enables biofilm formation (54). Several virulence properties contribute to the pathogenic potential of the organism. Expressed cell wall adhesins, including the members of the agglutinin-like sequence family (Als) and hyphal wall protein (Hwp1), are crucial for C. albicans attachment to host tissue and for multispecies biofilm formation (44, 55, 56). C. albicans secretes a number of hydrolytic enzymes, such as lipases, esterases, and secreted aspartyl proteinases (Saps), that affect a variety of processes, including biofilm formation with streptococci, tissue invasion, and immune evasion (57–59). A newly reported candidalysin, a cytolytic peptide toxin secreted by C. albicans hyphae, causes damage to oral epithelial cells by intercalation, permeabilization, and calcium influx; triggers a proinflammatory signaling pathway response; and activates epithelial immunity (60).

The oral carriage rate of C. albicans in healthy subjects ranges from 25% to 60% (43). Successful colonizers of the oral microbiota generally interact synergistically with one another (61). P. gingivalis, for example, can attach to S. gordonii and accumulate into heterotypic communities, a process facilitated by several adhesin-receptor interactions and phosphotyrosine-dependent signaling within P. gingivalis (12, 62). Similarly, C. albicans is usually present in heterotypic communities (16) and interacts synergistically with a variety of other organisms, including the oral streptococci (17, 19, 63). C. albicans can be isolated from periodontal pockets along with P. gingivalis (64, 65). In this study, we found that P. gingivalis and C. albicans can coadhere, both in suspension and in sessile communities, and that interspecies binding in both contexts is mediated by the InlJ internalin-family protein on the surface of P. gingivalis interacting with the candidal Als3 hyphal protein.

Internalins belong to a multigene family characterized by variable numbers of leucine-rich repeats (LRRs). The internalins InlA and InlB in *Listeria* are major virulence factors and mediate attachment and bacterial uptake by nonprofessional phagocytic epithelial cells (66). InlJ comprises a distinct class of internalins, and the LRR consensus sequence contains 21 leucine residues, compared to the standard 22 residues found in other internalins. In addition, a hydrophobic residue in one of the LRRs is replaced by a cysteine in InlJ, and InlJ thus possesses a total of 14 cysteine residues (67). In *Listeria*, InlJ is a sortase-LPXTG anchored adhesin upregulated during infection *in vivo* (68). Listerial InlJ can bind to MUC2 (the major component of intestinal mucus) and to a variety of human cells *in vitro* (68, 69), and oral infection with an *inlJ* mutant results in reduced *Listeria* levels in the intestine, mesenteric lymph nodes, liver, and spleen (70). In P. gingivalis, the InlJ homologue is upregulated following contact with gingival epithelial cells (71), and an *inlJ* mutant is deficient in homotypic biofilm formation by P. gingivalis (41). In the current study, we found that InlJ was required for maximal attachment of P. gingivalis to the hyphae of C. albicans. In addition, soluble recombinant InlJ protein was able to compete with whole P. gingivalis cells for attachment to *Candida*. These results establish a new role for InlJ of P. gingivalis as an adhesin mediating attachment to hyphae of C. albicans. In addition, an interdomain binding function extends the repertoire of internalin-family activities.

In order to identify the C. albicans receptor for P. gingivalis, we first tested the involvement of the Als3 hyphal protein. C. albicans defective for Als3 showed a significantly reduced ability to bind P. gingivalis. The ability of recombinant InlJ and of P. gingivalis expressing InlJ to bind to S. cerevisiae strains expressing Als3 corroborated the role of Als3 in mediating attachment through interactions with InlJ. Als3 is able to bind extracellular matrix (ECM) proteins and epithelial and endothelial cells (44, 72–74), induce endocytosis through adherence to E- or N-cadherins (75), and mediate trafficking to the brain (76). Als3 also mediates attachment to S. gordonii through binding to the SspA/B streptococcal surface proteins, and this interaction stimulates the development of a mixed bacterium-fungus community with a potentially increased risk for candidiasis (43, 77). Interestingly, P. gingivalis also bound to the SspA/B proteins of S. gordonii (78–80), raising the possibility that P. gingivalis and C. albicans could compete for binding to a streptococcal substratum.

To further delineate the role of the P. gingivalis InlJ in the interaction with C. albicans, a global transcriptional approach was undertaken. Here, 381 P. gingivalis genes were shown to be InlJ regulated, among which 153 (79 with a Log2 fold change >1) were unique to coincubation with C. albicans. While this level of community-dependent regulation is similar to data reported for S. gordonii in association with C. albicans (81), the differentially expressed genes were functionally different, indicating organism-specific responses of bacteria to *Candida*. Overall, GO analysis indicated that InlJ may play a role in increased growth and cellular division during coculture. Indeed, these analyses showed that peptidoglycan biosynthesis potential was increased during coculture, a characteristic demonstrated within complex polymicrobial biofilms from periodontitis patients (82). Alternatively, the close association between the organisms mediated by InlJ may facilitate generation of an anaerobic environment by C. albicans which enhances the growth of P. gingivalis, as has been demonstrated with *Candida* and *Bacteroides* species (83). Perhaps the most notable cluster of coassociated subnetworks of genes that were uniquely upregulated in coculture were those from the T9SS, which is widely distributed in the *Fibrobacteres-Chlorobi-Bacteroidetes* superphylum and secretes cargo proteins that are often cell associated and possess a conserved C-terminal domain (48). Many of the substrate proteins are considered major virulence factors in P. gingivalis, including the gingipains and other proteases; peptidylarginine deiminase (PAD), which catalyzes the conversion of peptidylarginine to peptidyl citrulline; and InlJ itself. Upregulation of T9SS components in P. gingivalis-C. albicans communities is thus consistent with elevated community pathogenicity, or nososymbiocity (11). Similarly, communities of P. gingivalis with the accessory pathogen S. gordonii, which are synergistically pathogenic (13), show an increase in expression of genes encoding T9SS components (84). Periodontal diseases are polymicrobial infections, and it is the heterotypic community that is considered the fundamental unit of pathogenicity (61). As an inhabitant of these complex multispecies biofilms, P. gingivalis, which is a keystone pathogen, may thus have evolved mechanisms to sense the community environment and respond through upregulation of the secretion system which can modulate virulence potential.

## MATERIALS AND METHODS

### Microbial strains and growth conditions.

The bacterial and yeast strains used in this study are listed in Table 1. P. gingivalis strain ATCC 33277 and its isogenic mutants Δ*inlJ*, Δ*fimA*, 33277+pT-COW, and cΔ*inlJ* (see below) were cultured in Trypticase soy broth (TSB) supplemented with yeast extract (1 mg/ml), hemin (5 µg/ml) and menadione (1 µg/ml) (TSBHM). Erythromycin (5 µg/ml) or tetracycline (1 µg/ml) were incorporated into the medium for the growth of strains Δ*inlJ*, Δ*fimA*, cΔ*inlJ*, and 33277+pT-COW as appropriate. C. albicans strains were maintained aerobically on Sabouraud dextrose agar at 37°C, and broth cultures were grown in YPD broth (1% yeast extract, 2% neopeptone, 2% glucose) at 37°C with shaking. YPT medium (yeast nitrogen base, 10 mM NaH_2_PO_4_ buffer [pH 7.0], 0.05% Bacto tryptone) supplemented with 0.4% glucose (YPT-Glu) was utilized to support C. albicans biofilm formation and induction of hyphae. S. cerevisiae cells were cultured with shaking at 30°C in complete synthetic medium (CSM) supplemented with 0.67% yeast nitrogen base and 2% glucose (CSM-Glu). Escherichia coli strains were grown aerobically with shaking at 37°C in Luria-Bertani broth supplemented with ampicillin (100 µg/ml) when required.

**TABLE 1:** Microbial strains used in this study

Strain	Characteristic^a^	Source or reference
P. gingivalis		
33277	Wild type	Laboratory collection
33277 Δ*fimA*	*fimA*-deficient mutant *fimA*::*tet*	87
33277 Δ*inlJ*	*inlJ*-deficient mutant *inlJ*::*erm*	41
33277 cΔ*inlJ*	*inlJ*-deficient mutant *inlJ*::*erm* with plasmid Pt-COW:*inlJ* expressing InlJ protein	This study
33277+pTCOW	P. gingivalis 33277 with pT-COW plasmid	This study
		
C. albicans		
SC5314	Wild type	88
UB1936	*iro1*-*ura3*Δ::*λimm*^*434*^/*iro1*Δ-*ura3*Δ::*λimm*^*434*^/Clp10; CAI-4/Clp10 parent strain	90
UB1930	*als3lg*Δ/*als3sm*Δ*-URA3*; deficient in Als3	74
		
S. cerevisiae		
UB2155	pB542; BY4742 containing Gateway destination vector pBC542 (8.3 kb; Ap^r^; pMB1 *ori*)	89
UB2156	pBC542-*als3sm*; BY4742 expressing C. albicans Als3sm (small allele)	89
UB2157	pBC542-*als3lg*; BY4742 expressing C. albicans Als3lg (large allele)	89
UB2161	pBC542-*cwp1*; BY4742 expressing S. cerevisiae Cwp1	89
		
E. coli		
TOP10	F^−^ *mrcA* Δ(*mrr-hsdRMS*-*mcrBC*) Φ80*lacZ*ΔM15 Δ*lacX74 recA1 araD139* Δ(*ara leu*)*7697 galU galK rpsL* (Str^r^) *endA1 nupG*	Invitrogen

a Ap^r^, ampicillin resistance; Str^r^, streptomycin resistance.

### Complementation of strain Δ*inlJ*.

For complementation of the *ΔinlJ* mutant, the DNA sequence containing the promoter and the coding region of *inlJ* was amplified from P. gingivalis 33277 chromosomal DNA using primers F1 (AATAGGATCCGTCCCGACTTTCCGATATATAAG) (containing a BamHI restriction site) and R2 (AATAGTCGACTTACGGCATCGCGGTTTTG) (containing a SalI restriction site). The shuttle vector pT-COW plasmid was digested with the appropriate restriction enzymes to allow cloning of the amplified PCR product into the *tetC* region. The resulting plasmid, pT-COW:*inlJ*, was transformed into E. coli TOP10 and selected with ampicillin. Purified pT-COW:*inlJ* was introduced into the Δi*nlJ* strain by conjugation as described previously (85). The presence of the pT-COW:*inlJ* plasmid and of the *ermF* gene on the chromosome of the transconjugants was confirmed by PCR and sequencing. The resulting strain was designated cΔ*inlJ*. As determined by quantitative reverse transcription-PCR (qRT-PCR), the expression levels of the *inlJ* gene were similar (*P* > 0.05) in strains cΔ*inlJ* and 33277.

### Expression of recombinant InlJ protein (rInlJ).

InlJ protein was expressed as a His-tagged fusion protein using an Expressway cell-free E. coli expression system (Invitrogen, Carlsbad, CA). Briefly, the entire coding region of *inlJ* (PGN_1611) was amplified from a P. gingivalis 33277 genomic template using primers F1 (ATGAAAAGAAAACCGCTATTCTCAG) and R1 (TTACGGCATCGCGGTTTTGATCG), cloned into pEXP5-NT/TOPO, and transformed into E. coli TOP10 cells. Following confirmation by sequencing, soluble His-tagged protein was obtained using MagneHis particles (GE Healthcare, Pittsburgh, PA). The purity of the resulting protein was verified by SDS-PAGE electrophoresis.

### C. albicans-P. gingivalis interactions in the planktonic phase.

Binding interactions between C. albicans and P. gingivalis in suspension were measured essentially as described previously (43). C. albicans cells were grown for 16 h in YPD medium, harvested by centrifugation (4,000 × *g*, 10 min), washed twice with YPT medium, and suspended at an optical density at 600 nm (OD_600_) of 1.0 (~1 × 10^7^ cells/ml). Aliquots (0.2 ml) of cell suspension were then incubated in YPT-Glu (1.8 ml) at 37°C for 2 h with shaking to induce formation of hyphae. P. gingivalis was cultured for 16 h in TSBHM medium, harvested by centrifugation (4,000 × *g*, 10 min), washed with phosphate-buffered saline (PBS), suspended in 1.5 mM fluorescein isothiocyanate (FITC) solution, and incubated at 20°C for 30 min. After two washes with PBS to remove excess FITC, cells were suspended in YPT-Glu supplemented with hemin (5 µg/ml) and menadione (1 µg/ml) (YPT-GluHM) at an OD_600_ of 0.5. FITC-labeled bacteria were then added to the yeast cell suspension and incubated for 1 h at 37°C with shaking. Samples (50 µl) of the suspension were applied to microscope slides and visualized by light and fluorescence microscopy. Images were analyzed using Zeiss Zen imaging software. Attachment of P. gingivalis to C. albicans was categorized as representing either “binding” (extensive attachment of bacteria to hyphae with bacterial clumping and bacterial cells aligned along hyphae in distinct patches) or “no binding” (sparse or no interactions between bacteria and hyphae) (see Fig. S3 in the supplemental material). The numbers of hyphae within these categories were expressed as the percentages of the total number of hyphae counted from 4 independent experiments. One hundred hyphal cells were counted for each assay. For inhibition assays, C. albicans was incubated with rInlJ or control protein at 37°C for 2 h, prior to addition of P. gingivalis.

10.1128/mBio.00202-18.3 FIG S3  Example of images used to calculate binding of P. gingivalis to *Candida* hyphae. Fluorescently (FITC) labeled P. gingivalis cells were incubated with C. albicans hypha-forming cells for 1 h. Binding was visualized by phase-contrast microscopy (upper panels) and by fluorescence microscopy (lower panels). Download FIG S3, EPS file, 1.8 MB. Copyright © 2018 Sztukowska et al. 2018 Sztukowska et al. This content is distributed under the terms of the Creative Commons Attribution 4.0 International license.

### Dual-species biofilm formation.

Biofilm formation by C. albicans and P. gingivalis was assayed as described previously (43). Sterile glass coverslips were incubated with filter-sterilized 10% saliva for 16 h at room temperature and washed twice with PBS. C. albicans cells were grown for 16 h in YPD medium, harvested by centrifugation (4,000 × *g*, 10 min), washed twice with YPT medium, and suspended to an OD_600_ of 1.0. Cells (1 × 10^6^) were added to wells of 12-well plates containing saliva-coated coverslips and YPT-Glu medium (0.9 ml) and were incubated at 37°C for 2 h with gentle shaking to induce formation of hyphae. The YPT-Glu medium was replaced with YPT-GluHM, and FITC-labeled P. gingivalis cells (5 × 10^6^) were added. Dual-species cultures were incubated for a further 1 h at 37°C with gentle shaking (50 rpm). Unbound bacteria in suspension were removed, and 1 ml of YPT medium containing hexidium iodide (Sigma-Aldrich, St. Louis, MO) was added for 5 min to fluorescently stain C. albicans. Coverslips were washed twice with PBS, mounted with Prolong Gold (Invitrogen), and imaged with a Leica SP8 confocal microscope. Images were analyzed using Volocity 6.3 software (PerkinElmer, Waltham, MA). For inhibition assays, C. albicans was incubated with rInlJ or control protein at 37°C for 2 h, prior to addition of P. gingivalis.

### Whole-cell enzyme-linked immunosorbent assay (ELISA).

S. cerevisiae cells were grown for 16 h in CSM-Glu medium, harvested by centrifugation (4,000 × *g*, 10 min), washed twice in PBS, and suspended at an OD_600_ of 1.0. Microtiter plates were coated with 100 µl of cell suspension at room temperature for 1 h. The coated plate was washed twice with 0.1% Tween–PBS followed by blocking performed for 1 h with 100 µl of 10% skim milk–PBS and was further washed as described above. S. cerevisiae cells were then reacted for 1 h with either rInlJ (5 µg) or P. gingivalis cells (1 × 10^7^). After a washing step, bound rInlJ protein was detected using a 1:2,000 dilution of His-tagged monoclonal antibody (MAb) (Cell Signaling, Inc., Danvers, MA) and P. gingivalis was detected with P. gingivalis whole-cell antibodies (1:10,000). After 1 h of incubation, reactions were developed with goat anti-rabbit horseradish peroxidase (HRP)-conjugated IgG (Cell Signaling) and TMB substrate (Invitrogen). The reaction was stopped with 100 µl of 1 N HCl, and the OD value was determined at 450 nm.

### RNA sequencing (RNA-Seq).

C. albicans was induced to form hyphae in YPT-GluHM for 2 h, as described above. Equal numbers of P. gingivalis and C. albicans cells (5 × 10^8^) were then incubated together in the planktonic phase in YPT-GluHM for 1 h. The two species were treated identically in monocultures in separate experiments. Cells were harvested by centrifugation and suspended in ice-cold RLT buffer (Qiagen, Manchester, United Kingdom) containing 2-mercaptoethanol. Acid-washed Biospec glass beads (0.6 ml) were added, and cells were disrupted by alternating shaking (30 s) using a FastPrep-25 bead beater (MP Biomedicals, Santa Ana, CA) and incubating for 1 min on ice (repeated 3 times). RNA was extracted and purified using an RNeasy minikit (Qiagen) with on-column DNase digestion (Qiagen). rRNA was depleted with a RiboZero Magnetic Gold kit (Epicentre, Illumina Inc., Madison, WI), and lllumina sequencing libraries were prepared using ScriptSeq v2 (Epicentre) with 10 cycles of PCR amplification. Paired-end sequencing of 100 bp was undertaken using a HiSeq 2500 system (Illumina) in high-output mode with Truseq v3 reagents.

FASTQ data were filtered using the fastq-mcf command from the EA-UTILS suite to remove adapter sequences and low-quality bases (86). Filtered data were aligned against the reference using Bowtie v.2.2.6. The resulting aligned reads were processed with SAMtools (v0.1.19), and gene features were counted using SAM files and the function htseq-count from Python package HTSeq v0.9.1 (https://pypi.python.org/pypi/HTSeq). The DESeq2 package was then used to apply a negative binomial model to account for dispersion between samples, before assessing differential expression between variables. *P* values were calculated using DESeq2, and Benjamini-Hochberg adjusted *P* values of <0.05 were considered significant. Following identification of differentially expressed P. gingivalis genes, unique genes (with an adjusted *P* value of <0.05) that were differentially expressed only during the coincubation with C. albicans were discerned. P. gingivalis genes were annotated with their associated Gene Ontology identifiers from UniProt (http://www.uniprot.org/). GO enrichment/overrepresentation analysis was performed by the use of the R GOSeq package, which implements a Wallenius hypergeometric distribution to account for bias based on gene length. Protein interaction networks were drawn from significantly differentially expressed genes using STRING (https://string-db.org/cgi/input.pl).
